# (η^5^-Penta­methyl­cyclo­penta­dien­yl)(η^6^-toluene)­ruthenium(II) hexa­fluorido­phosphate

**DOI:** 10.1107/S1600536810036299

**Published:** 2010-09-15

**Authors:** Wylie W. N. O, Alan J. Lough, Robert H. Morris

**Affiliations:** a Department of Chemistry, University of Toronto, Toronto, Ontario, Canada M5S 3H6

## Abstract

In the title complex, [Ru(C_7_H_8_)(C_10_H_15_)]PF_6_, the cation lies on a mirror plane and the anion lies on an inversion center. The distance between the Ru atom and the centroid of the benzene ring is 1.706 (5) Å and the distance between the Ru atom and the cyclo­penta­dienyl ring is 1.811 (5) Å. The crystal structure is stabilized by weak C—H⋯F hydrogen bonds. The H atoms of the methyl groups which lie on the mirror plane are disordered over two sites with equal occupancies.

## Related literature

For reviews on half-sandwich complexes containing group 8 metals, see: Coville *et al.* (1992 ▶); Jiménez-Tenorio *et al.* (2004 ▶). For the synthesis and properties of the title complex, see: Arliguie *et al.* (1988 ▶); Schmid *et al.* (2003 ▶); Loughrey *et al.* (2008 ▶). For related structures, see: Fagan *et al.* (1989 ▶, 1990 ▶); He *et al.* (1991 ▶); Nolan *et al.* (1993 ▶). For bifunctional catalysts for the homogenous hydrogenation of polar bonds, see: Clapham *et al.* (2004 ▶); O *et al.* (2010 ▶).
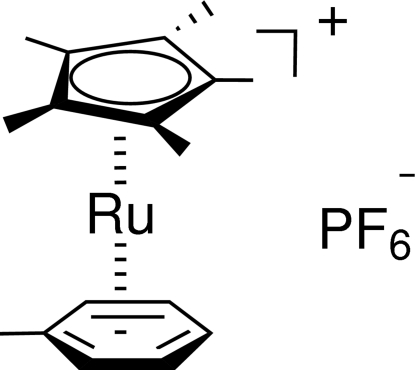


## Experimental

### 

#### Crystal data

[Ru(C_7_H_8_)(C_10_H_15_)]PF_6_
*M* *_r_* = 473.39
Orthorhombic, 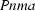

*a* = 13.9735 (4) Å
*b* = 15.3266 (4) Å
*c* = 8.6576 (6) Å
*V* = 1854.17 (15) Å^3^
*Z* = 4
Mo *K*α radiation
μ = 0.99 mm^−1^
*T* = 150 K
0.22 × 0.15 × 0.10 mm

#### Data collection

Nonius KappaCCD diffractometer
Absorption correction: multi-scan (*SORTAV*; Blessing, 1995 ▶) *T*_min_ = 0.711, *T*_max_ = 0.863
11870 measured reflections
2200 independent reflections
1611 reflections with *I* > 2σ(*I*)
*R*_int_ = 0.062

#### Refinement

*R*[*F* ^2^ > 2σ(*F* ^2^)] = 0.048
*wR*(*F* ^2^) = 0.131
*S* = 1.07
2200 reflections
128 parameters
H-atom parameters constrained
Δρ_max_ = 2.11 e Å^−3^
Δρ_min_ = −2.04 e Å^−3^

### 

Data collection: *COLLECT* (Nonius, 2002 ▶); cell refinement: *DENZO-SMN* (Otwinowski & Minor, 1997 ▶); data reduction: *DENZO-SMN*; program(s) used to solve structure: *SIR92* (Altomare *et al.*, 1994 ▶); program(s) used to refine structure: *SHELXTL* (Sheldrick, 2008 ▶); molecular graphics: *PLATON* (Spek, 2009 ▶); software used to prepare material for publication: *SHELXTL*.

## Supplementary Material

Crystal structure: contains datablocks global, I. DOI: 10.1107/S1600536810036299/pk2266sup1.cif

Structure factors: contains datablocks I. DOI: 10.1107/S1600536810036299/pk2266Isup2.hkl

Additional supplementary materials:  crystallographic information; 3D view; checkCIF report

## Figures and Tables

**Table 1:** Hydrogen-bond geometry (Å, °)

*D*—H⋯*A*	*D*—H	H⋯*A*	*D*⋯*A*	*D*—H⋯*A*
C2—H2*A*⋯F2^i^	1.00	2.46	3.450 (4)	173
C2—H2*A*⋯F3^i^	1.00	2.54	3.243 (5)	127
C3—H3*A*⋯F2^ii^	1.00	2.44	3.356 (5)	151
C8—H8*C*⋯F3^iii^	0.98	2.55	3.258 (5)	129
C10—H10*B*⋯F1^iv^	0.98	2.54	3.515 (6)	175

Symmetry codes: (i) 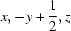; (ii) 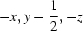; (iii) 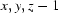; (iv) 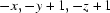.

**Fig. 1.**
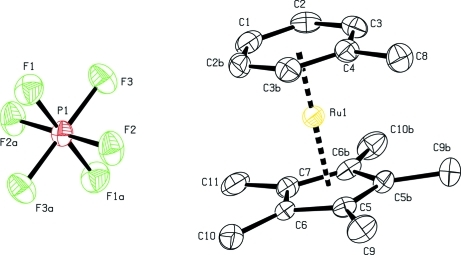
The molecular structure of the title compound showing 30% probability ellipsoids. Atoms labeled with suffixes 'a' and 'b' are related by the symmetry codes (-x, -y + 1, -z + 1) and (x, -y + 1/2, z) respectively.

**Fig. 2.**
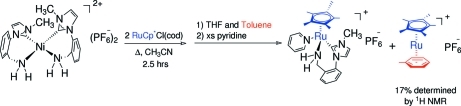
The reaction scheme.

## supplementary crystallographic information

### Comment

The heterolytic splitting of dihydrogen across a transition metal-amido bond provides an important metal-hydride and a protic amine group for the efficient catalytic homogenous hydrogenation of polar bonds to produce valuable alcohols and amines (Clapham *et al.*, 2004). We are interested in the use of chelating primary amine and N-heterocyclic carbene ligands (C—NH_2_) that resemble those of the phosphine-amine analogues. Thus, the transmetalation reaction of 1.5 equiv of RuCp*(cod)Cl (cod = 1,5-cyclooctadiene) and [Ni(C—NH_2_)_2_] (PF_6_)_2_ in acetonitrile, and subsequent workup in tetrahydrofuran and excess pyridine afforded the active catalyst, [RuCp*(C—NH_2_)(py)]PF_6_ (Fig. 2), for the hydrogenation of polar bonds in basic solution (O *et al.*, 2010). The use of 2 equiv. of RuCp*(cod)Cl and 1 equiv of [Ni(C—NH_2_)_2_] (PF_6_)_2_, with subsequent workup in tetrahydrofuran, toluene and pyridine mixtures, however, afforded selective crystallization of small amounts of title molecule, [Cp*Ru(η^6^-toluene)]PF_6_, as a side product. We report here the crystal structure of the title molecule. The synthesis of such compounds have been reported elsewhere (Fagan *et al.*, 1989; Schmid *et al.*, 2003; Loughrey *et al.*, 2008). The spectroscopic data for the reaction mixture containing the title molecule matches those reported in the literature (Arliguie *et al.*, 1988; Loughrey *et al.*, 2008).

The molecular structure of the title complex is shown in Fig. 1. The title sandwich complex consists of a coordinated planar arene ring and a pentamethylcyclopentadienyl ring in η^6^– and η^5^– hapticities, respectively. The bond distances are in reasonable agreement for analogous complexes with, for example, coordinated hexamethylbenzene and anisole in η^6^– hapticities (Fagan *et al.*, 1989, 1990; He *et al.*, 1991; Nolan *et al.*, 1993). The distance between the Ru atom and the centroid of the benzene ring is 1.706 (5) Å and the distance between the Ru atom and the cyclopentadienyl ring is 1.811 (5) Å. The angle formed with the centroids of the coordinated rings and the Ru^II^ ion is 179.49 (15)°. The crystal structure is stabilized by weak C—H···F hydrogen bonds.

### Experimental

A Schlenk flask was charged with [Ni(C—NH_2_)_2_](PF_6_)_2_ (32 mg, 0.084 mmol) and RuCp*(cod)Cl (30 mg, 0.041 mmol). Dry acetonitrile (8 ml) was added to the reaction mixture, and it was refluxed under an argon atmosphere for 3 h. The deep green solution was evaporated under reduced pressure, and the residue was extracted with oxygen-free tetrahydrofuran (4 ml) and toluene (1 ml), and filtered through a pad of Celite under a nitrogen atmosphere. To the yellow-brown solution was added pyridine (11 mg, 15 fold excess), and the orange coloured solution was evaporated under reduced pressure. The solid residue was extracted with tetrahydrofuran (3 ml) and dichloromethane (1 ml). Addition of diethyl ether (8 ml) to this solution afforded an orange precipitate, which gave the crude products of [RuCp*(C—NH_2_)py]PF_6_ and about 17% of the title salt, [Cp*Ru(η^6^-toluene)]PF_6_, as determined by ^1^H NMR spectroscopy of the bulk solid. This was filtered and dried in vacuum to yield an orange powder. Suitable crystals for an X-ray diffraction study were obtained by slow diffusion of diethyl ether into a saturated solution of the mixture in acetone under a nitrogen atmosphere to afford colourless blocks.

### Refinement

Hydrogen atoms were placed in calculated positions with C—H distances ranging from 0.95 to 1.00 Å and included in the refinement in a riding-model approximation with *U*_iso_(H) = 1.2*U*_eq_(C) or *U*_iso_(H) = 1.5*U*_eq_(C) for methyl H atoms.

### Crystal data

**Table:**

[Ru(C_7_H_8_)(C_10_H_15_)]PF_6_	*F*(000) = 952
*M**_r_* = 473.39	*D*_x_ = 1.696 Mg m^−^^3^
Orthorhombic, *P**n**m**a*	Mo *K*α radiation, λ = 0.71073 Å
Hall symbol: -P 2ac 2n	Cell parameters from 11870 reflections
*a* = 13.9735 (4) Å	θ = 2.7–27.5°
*b* = 15.3266 (4) Å	µ = 0.99 mm^−^^1^
*c* = 8.6576 (6) Å	*T* = 150 K
*V* = 1854.17 (15) Å^3^	Block, colourless
*Z* = 4	0.22 × 0.15 × 0.10 mm

### Data collection

**Table:**

Nonius KappaCCD diffractometer	2200 independent reflections
Radiation source: fine-focus sealed tube	1611 reflections with *I* > 2σ(*I*)
graphite	*R*_int_ = 0.062
Detector resolution: 9 pixels mm^-1^	θ_max_ = 27.5°, θ_min_ = 2.7°
φ scans and ω scans with κ offsets	*h* = −17→18
Absorption correction: multi-scan (*SORTAV*; Blessing, 1995)	*k* = −15→19
*T*_min_ = 0.711, *T*_max_ = 0.863	*l* = −11→11
11870 measured reflections	

### Refinement

**Table:**

Refinement on *F*^2^	Primary atom site location: structure-invariant direct methods
Least-squares matrix: full	Secondary atom site location: difference Fourier map
*R*[*F*^2^ > 2σ(*F*^2^)] = 0.048	Hydrogen site location: inferred from neighbouring sites
*wR*(*F*^2^) = 0.131	H-atom parameters constrained
*S* = 1.07	*w* = 1/[σ^2^(*F*_o_^2^) + (0.065*P*)^2^ + 3.7819*P*] where *P* = (*F*_o_^2^ + 2*F*_c_^2^)/3
2200 reflections	(Δ/σ)_max_ = 0.003
128 parameters	Δρ_max_ = 2.11 e Å^−^^3^
0 restraints	Δρ_min_ = −2.03 e Å^−^^3^

### Special details

**Table:**

Geometry. All e.s.d.'s (except the e.s.d. in the dihedral angle between two l.s. planes) are estimated using the full covariance matrix. The cell e.s.d.'s are taken into account individually in the estimation of e.s.d.'s in distances, angles and torsion angles; correlations between e.s.d.'s in cell parameters are only used when they are defined by crystal symmetry. An approximate (isotropic) treatment of cell e.s.d.'s is used for estimating e.s.d.'s involving l.s. planes.
Refinement. Refinement of *F*^2^ against ALL reflections. The weighted *R*-factor *wR* and goodness of fit *S* are based on *F*^2^, conventional *R*-factors *R* are based on *F*, with *F* set to zero for negative *F*^2^. The threshold expression of *F*^2^ > σ(*F*^2^) is used only for calculating *R*-factors(gt) *etc*. and is not relevant to the choice of reflections for refinement. *R*-factors based on *F*^2^ are statistically about twice as large as those based on *F*, and *R*- factors based on ALL data will be even larger.

### Fractional atomic coordinates and isotropic or equivalent isotropic displacement parameters (Å^2^)

**Table:**

	*x*	*y*	*z*	*U*_iso_*/*U*_eq_	Occ. (<1)
Ru1	0.09419 (3)	0.2500	0.05036 (5)	0.02470 (18)	
C1	−0.0364 (4)	0.2500	0.1948 (8)	0.0364 (15)	
H1	−0.0414	0.2500	0.3042	0.044*	
C2	−0.0326 (3)	0.1709 (3)	0.1144 (5)	0.0329 (10)	
H2A	−0.0235	0.1153	0.1730	0.040*	
C3	−0.0226 (3)	0.1708 (3)	−0.0472 (5)	0.0318 (10)	
H3A	−0.0065	0.1147	−0.1005	0.038*	
C4	−0.0174 (4)	0.2500	−0.1323 (7)	0.0300 (13)	
C5	0.2303 (3)	0.2969 (3)	−0.0428 (5)	0.0266 (9)	
C6	0.2204 (3)	0.3259 (3)	0.1143 (5)	0.0266 (9)	
C7	0.2144 (4)	0.2500	0.2112 (7)	0.0267 (13)	
C8	−0.0027 (5)	0.2500	−0.3057 (7)	0.0393 (16)	
H8A	−0.0634	0.2363	−0.3571	0.059*	0.50
H8B	0.0452	0.2060	−0.3333	0.059*	0.50
H8C	0.0194	0.3077	−0.3389	0.059*	0.50
C9	0.2427 (3)	0.3550 (3)	−0.1801 (6)	0.0391 (11)	
H9A	0.3097	0.3733	−0.1875	0.059*	
H9B	0.2018	0.4065	−0.1689	0.059*	
H9C	0.2247	0.3232	−0.2739	0.059*	
C10	0.2220 (3)	0.4184 (3)	0.1676 (6)	0.0405 (12)	
H10A	0.2883	0.4368	0.1846	0.061*	
H10B	0.1860	0.4235	0.2644	0.061*	
H10C	0.1926	0.4557	0.0888	0.061*	
C11	0.2061 (4)	0.2500	0.3875 (8)	0.0399 (16)	
H11A	0.1816	0.3066	0.4224	0.060*	0.50
H11B	0.2692	0.2397	0.4331	0.060*	0.50
H11C	0.1620	0.2037	0.4200	0.060*	0.50
P1	0.0000	0.5000	0.5000	0.0282 (4)	
F1	−0.09922 (18)	0.5503 (2)	0.4824 (4)	0.0454 (7)	
F2	0.0208 (2)	0.51551 (18)	0.3204 (3)	0.0450 (7)	
F3	−0.0520 (2)	0.40978 (18)	0.4592 (3)	0.0435 (7)	

### Atomic displacement parameters (Å^2^)

**Table:**

	*U*^11^	*U*^22^	*U*^33^	*U*^12^	*U*^13^	*U*^23^
Ru1	0.0238 (3)	0.0249 (3)	0.0255 (3)	0.000	0.00149 (19)	0.000
C1	0.024 (3)	0.052 (4)	0.033 (4)	0.000	0.005 (3)	0.000
C2	0.026 (2)	0.034 (2)	0.039 (3)	−0.0065 (18)	−0.0021 (19)	0.011 (2)
C3	0.028 (2)	0.031 (2)	0.036 (3)	−0.0049 (18)	−0.0049 (18)	−0.002 (2)
C4	0.026 (3)	0.038 (4)	0.025 (3)	0.000	−0.004 (2)	0.000
C5	0.023 (2)	0.029 (2)	0.028 (2)	−0.0033 (17)	0.0042 (16)	0.0022 (18)
C6	0.0190 (19)	0.033 (2)	0.028 (2)	−0.0030 (17)	0.0053 (16)	−0.0073 (18)
C7	0.021 (3)	0.040 (3)	0.019 (3)	0.000	−0.001 (2)	0.000
C8	0.042 (4)	0.052 (4)	0.024 (3)	0.000	−0.003 (3)	0.000
C9	0.041 (3)	0.042 (3)	0.035 (3)	−0.001 (2)	0.007 (2)	0.012 (2)
C10	0.037 (2)	0.036 (3)	0.049 (3)	−0.005 (2)	0.011 (2)	−0.011 (2)
C11	0.025 (3)	0.046 (4)	0.049 (4)	0.000	0.011 (3)	0.000
P1	0.0331 (8)	0.0276 (8)	0.0240 (8)	0.0019 (6)	0.0018 (7)	−0.0006 (7)
F1	0.0403 (16)	0.0456 (18)	0.0502 (18)	0.0127 (12)	−0.0027 (13)	−0.0017 (14)
F2	0.0653 (18)	0.0425 (16)	0.0273 (14)	−0.0008 (14)	0.0050 (13)	−0.0005 (13)
F3	0.0556 (18)	0.0311 (15)	0.0438 (17)	−0.0070 (13)	−0.0045 (13)	−0.0018 (12)

### Geometric parameters (Å, °)

**Table:**

Ru1—C7	2.181 (5)	C6—C7	1.436 (5)
Ru1—C6	2.184 (4)	C6—C10	1.491 (6)
Ru1—C6^i^	2.184 (4)	C7—C6^i^	1.436 (5)
Ru1—C5	2.187 (4)	C7—C11	1.531 (9)
Ru1—C5^i^	2.187 (4)	C8—H8A	0.9800
Ru1—C3	2.203 (4)	C8—H8B	0.9800
Ru1—C3^i^	2.203 (4)	C8—H8C	0.9800
Ru1—C1	2.211 (6)	C9—H9A	0.9800
Ru1—C2	2.217 (4)	C9—H9B	0.9800
Ru1—C2^i^	2.217 (4)	C9—H9C	0.9800
Ru1—C4	2.220 (6)	C10—H10A	0.9800
C1—C2	1.398 (6)	C10—H10B	0.9800
C1—C2^i^	1.398 (6)	C10—H10C	0.9800
C1—H1	0.9500	C11—H11A	0.9800
C2—C3	1.406 (6)	C11—H11B	0.9800
C2—H2A	1.0000	C11—H11C	0.9800
C3—C4	1.422 (6)	P1—F1^ii^	1.594 (3)
C3—H3A	1.0000	P1—F1	1.594 (3)
C4—C3^i^	1.422 (6)	P1—F2^ii^	1.599 (3)
C4—C8	1.515 (9)	P1—F2	1.599 (3)
C5—C6	1.437 (6)	P1—F3^ii^	1.601 (3)
C5—C5^i^	1.437 (9)	P1—F3	1.601 (3)
C5—C9	1.495 (6)		
			
C7—Ru1—C6	38.42 (14)	C2—C3—Ru1	72.0 (2)
C7—Ru1—C6^i^	38.42 (14)	C4—C3—Ru1	71.9 (3)
C6—Ru1—C6^i^	64.3 (2)	C2—C3—H3A	118.9
C7—Ru1—C5	64.28 (17)	C4—C3—H3A	118.9
C6—Ru1—C5	38.40 (15)	Ru1—C3—H3A	118.9
C6^i^—Ru1—C5	64.29 (15)	C3^i^—C4—C3	117.3 (6)
C7—Ru1—C5^i^	64.28 (17)	C3^i^—C4—C8	121.3 (3)
C6—Ru1—C5^i^	64.29 (15)	C3—C4—C8	121.3 (3)
C6^i^—Ru1—C5^i^	38.40 (15)	C3^i^—C4—Ru1	70.6 (3)
C5—Ru1—C5^i^	38.4 (2)	C3—C4—Ru1	70.6 (3)
C7—Ru1—C3	144.60 (13)	C8—C4—Ru1	127.7 (4)
C6—Ru1—C3	171.56 (16)	C6—C5—C5^i^	108.0 (2)
C6^i^—Ru1—C3	113.71 (17)	C6—C5—C9	125.4 (4)
C5—Ru1—C3	133.16 (16)	C5^i^—C5—C9	126.5 (3)
C5^i^—Ru1—C3	108.77 (17)	C6—C5—Ru1	70.7 (2)
C7—Ru1—C3^i^	144.60 (13)	C5^i^—C5—Ru1	70.81 (11)
C6—Ru1—C3^i^	113.71 (17)	C9—C5—Ru1	126.1 (3)
C6^i^—Ru1—C3^i^	171.56 (16)	C5—C6—C7	107.9 (4)
C5—Ru1—C3^i^	108.77 (17)	C5—C6—C10	125.8 (4)
C5^i^—Ru1—C3^i^	133.16 (16)	C7—C6—C10	126.2 (4)
C3—Ru1—C3^i^	66.9 (2)	C5—C6—Ru1	70.9 (2)
C7—Ru1—C1	105.9 (2)	C7—C6—Ru1	70.7 (3)
C6—Ru1—C1	121.52 (17)	C10—C6—Ru1	126.6 (3)
C6^i^—Ru1—C1	121.52 (17)	C6^i^—C7—C6	108.1 (5)
C5—Ru1—C1	157.77 (14)	C6^i^—C7—C11	125.9 (2)
C5^i^—Ru1—C1	157.77 (14)	C6—C7—C11	125.9 (2)
C3—Ru1—C1	66.77 (19)	C6^i^—C7—Ru1	70.9 (3)
C3^i^—Ru1—C1	66.77 (19)	C6—C7—Ru1	70.9 (3)
C7—Ru1—C2	117.11 (16)	C11—C7—Ru1	125.3 (4)
C6—Ru1—C2	150.82 (17)	C4—C8—H8A	109.5
C6^i^—Ru1—C2	106.93 (16)	C4—C8—H8B	109.5
C5—Ru1—C2	165.19 (17)	H8A—C8—H8B	109.5
C5^i^—Ru1—C2	127.41 (17)	C4—C8—H8C	109.5
C3—Ru1—C2	37.09 (17)	H8A—C8—H8C	109.5
C3^i^—Ru1—C2	78.75 (17)	H8B—C8—H8C	109.5
C1—Ru1—C2	36.81 (14)	C5—C9—H9A	109.5
C7—Ru1—C2^i^	117.11 (16)	C5—C9—H9B	109.5
C6—Ru1—C2^i^	106.93 (16)	H9A—C9—H9B	109.5
C6^i^—Ru1—C2^i^	150.82 (17)	C5—C9—H9C	109.5
C5—Ru1—C2^i^	127.41 (17)	H9A—C9—H9C	109.5
C5^i^—Ru1—C2^i^	165.19 (17)	H9B—C9—H9C	109.5
C3—Ru1—C2^i^	78.75 (17)	C6—C10—H10A	109.5
C3^i^—Ru1—C2^i^	37.09 (17)	C6—C10—H10B	109.5
C1—Ru1—C2^i^	36.81 (14)	H10A—C10—H10B	109.5
C2—Ru1—C2^i^	66.3 (2)	C6—C10—H10C	109.5
C7—Ru1—C4	174.3 (2)	H10A—C10—H10C	109.5
C6—Ru1—C4	138.37 (15)	H10B—C10—H10C	109.5
C6^i^—Ru1—C4	138.37 (15)	C7—C11—H11A	109.5
C5—Ru1—C4	110.35 (18)	C7—C11—H11B	109.5
C5^i^—Ru1—C4	110.35 (18)	H11A—C11—H11B	109.5
C3—Ru1—C4	37.51 (13)	C7—C11—H11C	109.5
C3^i^—Ru1—C4	37.51 (13)	H11A—C11—H11C	109.5
C1—Ru1—C4	79.8 (2)	H11B—C11—H11C	109.5
C2—Ru1—C4	67.48 (17)	F1^ii^—P1—F1	180.000 (1)
C2^i^—Ru1—C4	67.48 (17)	F1^ii^—P1—F2^ii^	89.60 (15)
C2—C1—C2^i^	120.1 (6)	F1—P1—F2^ii^	90.40 (15)
C2—C1—Ru1	71.8 (3)	F1^ii^—P1—F2	90.40 (15)
C2^i^—C1—Ru1	71.8 (3)	F1—P1—F2	89.60 (15)
C2—C1—H1	119.9	F2^ii^—P1—F2	180.000 (1)
C2^i^—C1—H1	119.9	F1^ii^—P1—F3^ii^	90.12 (16)
Ru1—C1—H1	128.7	F1—P1—F3^ii^	89.88 (16)
C1—C2—C3	120.1 (4)	F2^ii^—P1—F3^ii^	89.79 (14)
C1—C2—Ru1	71.4 (3)	F2—P1—F3^ii^	90.21 (14)
C3—C2—Ru1	70.9 (2)	F1^ii^—P1—F3	89.88 (16)
C1—C2—H2A	119.4	F1—P1—F3	90.12 (16)
C3—C2—H2A	119.4	F2^ii^—P1—F3	90.21 (14)
Ru1—C2—H2A	119.4	F2—P1—F3	89.79 (14)
C2—C3—C4	121.2 (4)	F3^ii^—P1—F3	180.0

Symmetry codes: (i) *x*, −*y*+1/2, *z*; (ii) −*x*, −*y*+1, −*z*+1.

### Hydrogen-bond geometry (Å, °)

**Table:**

*D*—H···*A*	*D*—H	H···*A*	*D*···*A*	*D*—H···*A*
C2—H2A···F2^i^	1.00	2.46	3.450 (4)	173
C2—H2A···F3^i^	1.00	2.54	3.243 (5)	127
C3—H3A···F2^iii^	1.00	2.44	3.356 (5)	151
C8—H8C···F3^iv^	0.98	2.55	3.258 (5)	129
C10—H10B···F1^ii^	0.98	2.54	3.515 (6)	175

Symmetry codes: (i) *x*, −*y*+1/2, *z*; (iii) −*x*, *y*−1/2, −*z*; (iv) *x*, *y*, *z*−1; (ii) −*x*, −*y*+1, −*z*+1.

## References

[bb1] Altomare, A., Cascarano, G., Giacovazzo, C., Guagliardi, A., Burla, M. C., Polidori, G. & Camalli, M. (1994). *J. Appl. Cryst.* **27**, 435.

[bb2] Arliguie, T., Chaudret, B., Jalon, F. & Lahoz, F. (1988). *Chem. Comm.* p. 998.

[bb3] Blessing, R. H. (1995). *Acta Cryst.* A**51**, 33–38. 10.1107/s0108767394005726 7702794

[bb4] Clapham, S. E., Hadzovic, A. & Morris, R. H. (2004). *Coord. Chem. Rev.* **248**, 2201–2237.

[bb5] Coville, N. J., Duplooy, K. E. & Pickl, W. (1992). *Coord. Chem. Rev.* **116**, 1–267.

[bb6] Fagan, P. J., Mahoney, W. S., Calabrese, J. C. & Williams, I. D. (1990). *Organometallics*, **9**, 1843–1852.

[bb7] Fagan, P. J., Ward, M. D. & Calabrese, J. C. (1989). *J. Am. Chem. Soc.* **111**, 1698–1719.

[bb8] He, X. D., Chaudret, B., Dahan, F. & Huang, Y.-S. (1991). *Organometallics*, **10**, 970–979.

[bb9] Jiménez-Tenorio, M., Puerta, M. C. & Valerga, V. (2004). *Eur. J. Inorg. Chem.* pp. 17–32.

[bb10] O, W. W. N., Lough, A. J. & Morris, R. H. (2010). *Chem. Commun.* In the press. 10.1039/c0cc02664f 20871933

[bb11] Loughrey, B. T., Healy, P. C., Parsons, R. G. & Williams, M. L. (2008). *Inorg. Chem.* **47**, 8589–8591. 10.1021/ic801159f 18783214

[bb12] Nolan, S. P., Martin, K. L., Buzatu, D., Trudell, M. L., Stevens, E. D. & Fagan, P. (1993). *J. Struct. Chem.* **4**, 367–375.

[bb13] Nonius (2002). *COLLECT* Nonius BV, Delft, The Netherlands.

[bb14] Otwinowski, Z. & Minor, W. (1997). *Methods in Enzymology*, Vol. 276, *Macromolecular Crystallography*, Part A, edited by C. W. Carter Jr & R. M. Sweet, pp. 307–326. New York: Academic Press.

[bb15] Schmid, A., Holger, P. & Lindel, T. (2003). *Eur. J. Inorg. Chem.* pp. 2255–2263.

[bb16] Sheldrick, G. M. (2008). *Acta Cryst.* A**64**, 112–122. 10.1107/S0108767307043930 18156677

[bb17] Spek, A. L. (2009). *Acta Cryst.* D**65**, 148–155. 10.1107/S090744490804362X PMC2631630 19171970

